# Mad Honey and the Poisoner King: A Case of Mass Grayanotoxin Poisoning in the Roman Military

**DOI:** 10.7759/cureus.38289

**Published:** 2023-04-29

**Authors:** Matthew D Turner

**Affiliations:** 1 Medicine, Madigan Army Medical Center, Lakewood, USA

**Keywords:** military history, mithridates, medical history, mad honey, roman, poisoning, grayanotoxin

## Abstract

We describe an episode of mass poisoning during the ancient Third Mithridatic War. In a brutal and well-planned ambush, forces from the Kingdom of Pontus destroyed a Roman column through the clever use of “mad honey.” Incapacitated by the acute poisoning, the Romans were unable to hold off the Pontic forces. We conclude that the debilitating symptoms that the Roman soldiers experienced were due to the presence of grayanotoxins in the honey. Although they were likely self-limiting, developing these symptoms in an enemy ambush made a lethal combination. The “mad honey” environmental threat continues to persist in the Black Sea region to this day and is an etiology that modern physicians should be aware of.

## Introduction and background

During the Third Mithridatic War, as the late Roman Republic expanded its control across northern Anatolia, Rome struggled against one of the fiercest and most cunning opponents it would ever face: King Mithridates Eupator VI of Pontus [1]. Mithridates was legendary among his contemporaries for his intelligence and fascination with pharmacology, specifically the use of poisons [2]. The war was a long and brutal one, and would ultimately end with the death of Mithridates and the fall of his kingdom to Roman imperialism [1].

However, Mithridates’ clever use of unconventional tactics, including poisons and primitive chemical weapons, won him several victories during the war, further contributing to his legend [1]. In this article, we discuss one of the most intriguing episodes of the conflict, where the Pontic forces successfully used poisonous “mad honey” against a Roman force.

## Review

The Third Mithridatic War

From 73 to 63 BC, the Roman Republic and King Mithridates VI of Pontus waged a bitter war that engulfed most of Asia Minor and much of the Eastern Mediterranean. The Third Mithridatic War, as it would come to be called, ultimately saw Mithridates’ defeat and the end of the Pontic Kingdom [1].

King Mithridates Eupator VI of Pontus is a fascinating figure. He was legendary for his memory and intelligence. Roman historians claimed that he could speak all 22 languages represented within his domain [2]. At his capital, he “surrounded himself with philosophers, physicians, and learned men,” and was known to send envoys across the Mediterranean to recruit famous foreign physicians [3]. He was in regular correspondence with several scientists, including Asclepiades of Bithynia, the founder of a medical school in Rome itself [1]. Perhaps due to the abundant natural resources of the Caucasus region, including many venomous snake species, the Pontic Kingdom had a long history of laying claim to poisons and magic [1]. It was the supposed home of the mythical witch Madea, creator of supernatural potions, and Mithridates’ grandfather, King Pharnaces I, had claimed to discover a “panacea” cure for ailments [1].

The king was fascinated with pharmacology, amassing an “international library of ethnobotanical and toxicological treatises” from sources that stretched from Mesopotamia to Gaul [1]. On one occasion, this directly saved his life - as he was hemorrhaging from a wound to his thigh on a battlefield, his physicians used snake venom to staunch the bleeding [1]. According to legend, he also “sought to harden himself against poisoning by taking increasing sub-lethal doses of those poisons of which he knew until he was able to tolerate lethal doses,” a process that to this day is known as *mithridatizing *[2]. In this regard, he was likely influenced by his childhood. His father was poisoned by his enemies in 120 BC, and there is evidence to suggest that he believed his mother, Queen Laodice, had attempted to poison him as a teenager [1]. Ultimately, Mithridates assassinated her first [4]. With the aid of his Greek botanist Kateuas, he seems to have successfully developed a tolerance to arsenic, routinely showing off his “remarkable ability to dine safely on poison-laced meat and wine” to awed dinner guests [1]. His extensive research into poisoning - sometimes on himself, sometimes on condemned criminals [5], sometimes via the assassination of “treacherous relatives and rivals” [1] - and botany eventually led to the creation of the *antidotum mithridaticum*, a “universal antidote” concocted out of a variety of ingredients [2]. Ironically, some scholars of the time claimed that, upon his final defeat, Mithridates attempted to commit suicide by poison, but “a long life of self-administered antidotes made him unable to fulfill his intention” [6]. In the end, his bodyguard had to finish the job [6]. Pompey eagerly ransacked the dead king’s archives and had several of the king’s documents regarding medicinal plants translated into Latin. Among the documents was the recipe for the legendary *antidotum mithridaticum*, which Pliny the Elder notes somewhat disappointingly “consisted of two dried walnuts, two figs, and twenty leaves of rue pounded together with a pinch of salt” [2]. Various incarnations of this recipe became popular in Rome, with Julius Caesar taking a modified form of it during his campaigns in Pontus 16 years later. Every Roman emperor after Nero took a daily dose of *mithridaticum *[1]. This antidote, eventually dubbed *theriaca*, was used in a slightly modified form up to the 19th century [5]. Even with this doubtful prescription, it is notable that Mithridates “enjoyed robust health into his 70s, at a time when the average lifespan was 45” [1].

Perhaps reflecting Mithridates’ interest in poisons, Pontic and Pontic-aligned forces used a number of unconventional tactics throughout the Third Mithridatic War. Roman legionnaires faced deadly poisoned arrows in open battle, had to contend with “wasps and wild beasts” released into their siege tunnels, and were even assaulted by the chemical weapon known as *maltha *- “burning, clinging naphtha from local petroleum pools” [7].

In 66 BC, the Roman politician general Gnaeus Pompeius, better known to the modern world as Pompey, used his enormous political influence to strong-arm the Roman Senate into passing the *Lex Manila*. This law granted him total control of the eastern Roman military and the glory that a final victory over Pontus would bring [8]. An extremely capable politician and general who secured Roman possessions on three continents and rose to the highest levels of power in the waning days of the Republic, if he had not been “eclipsed” by figures such as Julius Caesar, Pompey “could have lived in history as the Roman Empire’s mightiest architect” [9]. Pompey was known as Magnus, “the Great,” perhaps a nod to his “apparent physical resemblance” to Alexander the Great [10]. Ironically, his opponent Mithridates claimed descent from the legendary conqueror [1]. Always eager for glory, Pompey would exploit this particular trait throughout his military and political career. At the Triumph of 61 BC following his defeat of Mithridates, the historian Appian describes Pompey in a chariot, “one studded with gems, and he was wearing the cloak, it is said, of Alexander the Macedonian … he seems to have found it among the possessions of Mithridates…” [11]. At just 45 years old, this was the peak of his career, before he would later be outmaneuvered by later rivals such as Julius Caesar. As Beard notes, “He must have seemed unassailable - the Roman Alexander, an emperor in all but name” [11]. His victory in Pontus also brought him “immense wealth,” the command that the *Lex Manila* brought him gave him over 200,000,000 sesterces in land [12].

Upon Pompey’s arrival in Asia Minor, Mithridates sent an embassy to discuss terms of peace between the two powers. Unfortunately for the Pontic king, Pompey had no intention of securing an “embarrassingly easy victory” [8]. During the infamous Spartacus slave uprising, while generals such as Crassus “decisively crushed the revolt”, Pompey slaughtered 5,000 fugitives and claimed that it had been a massive victory over the rebellion [10]. This was a political miscalculation, for ever since, his detractors had claimed that he was a “vulture,” always ready to swoop in to steal credit for the hard work of others [13]. Pompey was not eager to repeat that mistake and desired a war that he could truthfully claim to be his own. Thus, he sent Mithridates impossible terms for unconditional surrender. The Pontus king inevitably rejected them, and Pompey’s campaign in Asia Minor began [8].

While Pompey did not display the military brilliance of men like Julius Caesar, he was an effective commander [9], and the war quickly turned in Roman’s favor, culminating in a total Roman victory in 63 BC [8]. Pompey’s campaign in Anatolia was not just one of conquest but also of colonization and subdividing the kingdom. The historian Strabo describes how Pompey established seven cities within Pontus, both providing settlements and dividing the conquered kingdom into “autonomous districts” that would provide “effective machinery for the collection of tribute due Rome,” a strategy that the Romans had recently used in their conquest of Spain [14]. The “municipalization” of the Pontic territory [14] made Mithridates’ kingdom little more than a memory. Pompey would find another 33 cities in the east to further secure Roman domains [11]. The former kingdom was soon integrated into the Roman sphere as part of the province of Bithynia-Pontus; this was particularly demeaning as Bithynia and Pontus had a long history of conflict [15].

The Heptacomitae

It was in this desperate setting of impending defeat that Pontus-aligned forces found themselves in during the Third Mithridatic War. In his magnum opus, *Geography*, Strabo of Amaseia (64 BC to 21 AD) [16] describes much of the conflict. A Roman citizen who was born in the province of Bithynia-Pontus shortly after Pompey’s conquest, Strabo had family members who had fought on both sides of the war [15]. *Geography *describes much of the devastation that remained in the Pontic landscape decades later [15].

In the third chapter of Book 12 of *Geography*, Strabo describes an incident during the Third Mithridatic War that involved the Heptacomitae, a “savage” people that dwelled in the Caucasus mountains, living on “the flesh of wild animals and on nuts” [17]. They appear to have been masters of asymmetrical warfare, often setting up complex “turrets” amid the overgrown forests of the mountains that allowed them to “attack wayfarers, leaping down upon them from their scaffolds” [17]. In 65 BC [7], the Heptacomitae, allies of Mithridates, “cut down three *maniples *of Pompey’s army when they passing through the mountainous country; for they mixed bowls of the crazing honey which is yielded by the tree-twigs, and placed them in the roads, and then, when the soldiers drank the mixture and lost their senses, they attacked them and easily disposed of them” [17]. The *maniple*, meaning “sheaf” or “bundle,” was the basic tactical unit of the Roman army in the early Republican era, and typically consisted of 180 men. However, by the time of the late Republic and Pompey’s invasion of Pontus, the *maniple *had almost entirely been replaced by the *cohort*, a larger unit that could be as large as 600 men [12]. It is unclear which unit of measurement Strabo is referring to here - Roman losses in this incident may have ranged from 480 men to as many as 1,800.

This was not the first time that an army in this region had been afflicted by the strange “mad honey” [16]. Three and a half centuries earlier, the Athenian military commander led his legendary “March of the Ten Thousand” through the modern Turkish province of Trabzon [16]. There, his men found an “astonishing” number of beehives [18]. Xenophon noted in his memoirs that “…the effect upon the soldiers who tasted the combs was, that they all went for the nonce quite off their heads, and suffered from vomiting and diarrhea, with a total inability to stand steady on their legs. A small dose produced a condition not unlike violent drunkenness, a large one an attack very like a fit of madness, and some dropped down, apparently at death’s door. So they lay, hundreds of them” [18]. Fortunately for Xenophon’s men, no enemies attacked, and within 24 hours even the most intoxicated soldier had “recovered their senses” [18]. Three to four days after initially consuming the honey, his men were recovered enough to “get on their legs again like convalescents after a severe course of medical treatment” and resume the march [18].

Even if Mithridates did not directly order the Heptacomitae to poison Pompey’s soldiers, he was almost certainly aware of the properties of “mad honey.” It may have been one of the ingredients that he routinely experimented with [1]. Some sources claim that it was Kateuas, his botanist, who proposed the mass poisoning [16,19], although Strabo makes no mention of this. Regardless, it would have been entirely in character for Mithridates, considered to be the master of ancient pharmacology [1], to order the deployment of such a weapon.

“Mad Honey” and grayanotoxins

“Mad honey” has been widely known among Turkey’s regions that border the Black Sea for millennia, and was considered to have medicinal properties by both the natives and the Romans [16]. In some areas of the Black Sea, it is still used to treat gastric disorders, hypertension, diabetes, and low libido [20], and can even be purchased in local markets as deli bal [21]. Pliny the Elder described it as having an “abnormally red color” with an unpleasant odor that “causes sneezing” [16]. It may also cause a “sharp burning sensation in the throat,” earning it the nickname “bitter honey” [20].

“Mad honey” is created due to the habits of local Caucasian bees, whose territory only covers an area of 5 km^2^. In the mountainous terrain of the Caucasus, each honey thus “contains only one valley’s flora” [20]. In Turkey, this flora includes six species of *Rhododendrons*, which, as members of the *Ericaceae *family, produce grayanotoxins in their nectar. Of these, *R. ponticum*, a “purple flowered evergreen shrub” [20] known as “mountain rose” for its extensive growth in mountainous areas [22], is the most notable due to the high concentration of grayanotoxins it produces [20].

Grayanotoxins are a class of neurotoxins that interfere with the sodium channels in cell membranes, preventing repolarization [20]. In a mild form, “mad honey” that has been formed from the collection of grayanotoxin-containing nectar may cause symptoms similar to that of a cholinergic response, with “dizziness, weakness, excessive perspiration, hypersalivation, nausea, vomiting and paresthesias” developing [20], consistent with Xenophon’s description [18]. Higher doses may lead to cardiac effects, including bradycardia, loss of consciousness [22], complete atrioventricular heart block [20], Wolff-Parkinson-White syndrome, shock [22], and even asystole [16]. The average dosage required for these symptoms depends on several factors, including the concentration of grayanotoxins and the host’s metabolism, but severe effects have been shown to develop from 5-30 g of “mad honey” [16]. For reference, the average spoonful of honey is approximately 15 g [16]. The symptoms have a rapid onset, ranging from several minutes to two or more hours, depending on the dose [22].

Remarkably, grayanotoxin poisoning is only rarely fatal, as the toxin is rapidly metabolized by the body. In the modern day, atropine sulfate is a sufficient treatment to alleviate the toxin’s bradycardic effects [22]. Even in untreated cases of severe intoxication, “the worst signs and symptoms last about 24 hours” [16], consistent with Xenophon’s description. Of note, the Athenian did not lose a single soldier to the toxin, despite his description of “hundreds” of men poisoned by it [18]. In periods as little as two to nine hours, patients will clinically improve, with a return of their heart rate and blood pressure to normal without any sort of intervention [22]. While poisonings still occur, particularly bordering the Black Sea and other areas with large concentrations of *Ericaceae *plants [23], no deaths have been documented since 1983 [20]. Similarly, a systematic review of 1,199 documented cases did not find a single fatality [21].

In this context, the Heptacomitae ambush must have required an impressive degree of timing and careful planning. Had the ambushers prepared the honey in too dense a concentration, the Roman soldiers would have likely developed symptoms within minutes, thus minimizing the number of men who ingested the honey. Preparing the honey with a more dilute dose of grayanotoxins would have ensured that more Romans ate the concoction, but would have simultaneously made it more difficult for an ambushing force to lay in wait without being detected while the toxin took its effect.

Even with the careful planning of the Heptacomitae, the failure of the Romans to detect the ruse seems remarkably foolish. “Mad honey” was a well-known medicinal compound, documented by both the Greek Aristotle [21] and the Roman Pliny the Elder listed it as medicine [16], and Pontus was legendary for its near-mythical use of poisons [1]. The Romans were no strangers to poisons themselves. Just a few years earlier, the Senate had passed the *Lex Cornelia de sicariis et veneficis* (“Cornelia Law on Assassins and Poisoners”) in a vain attempt to prevent the rash of poisonings in the city [4]. Even outside of assassinations and murders, mass poisoning during warfare was known to the Romans as well. Several decades prior the general Manius Aquillius had crushed an insurrection in Asia Minor by “pouring an unknown poison into the springs supplying the rebelling cities” [7]. In this context, a detachment of soldiers marching through enemy territory and consuming honey that they found in “bowls” alongside the road [17] demonstrates a level of military incompetence that would have been remarkably out of place for the highly disciplined and battle-hardened legions of the late Republic [12].

While it is possible that Strabo may have fabricated the story out of a sense of national pride for the fallen kingdom of his ancestors [15], these authors opine that it is unlikely. Under Mithridates, Pontus used a number of unconventional weapons throughout the Third Mithridatic War; using an already-established tactic would have been entirely in character for Pontic forces [7]. Strabo also “moved successfully among the political elite at Rome” [15]. It is difficult to see how fabricating a Roman defeat would have endeared him to the upper echelons of Roman society. Ultimately, it appears likely that the ambush occurred as Strabo described, perhaps due to a lapse in discipline or a simple human error on the Romans’ part.

## Conclusions

Although the Kingdom of Pontus successfully employed an unconventional tactic against the Roman invaders to devastating effect in this instance, it was not enough to turn the tide of the war. However, the mass poisoning of the Roman legionaries that Strabo describes in *Geography *is a fascinating milestone in both military and medical history. The “mad honey” phenomenon, due to the presence of grayanotoxins from local *R. ponticum* plants, was cleverly used in this ancient ambush. Although the poisoning is self-limiting in all but the most severe cases, the incapacitated Romans would have been helpless against Pontic forces. Although Mithridates may not have directly ordered the use of “mad honey,” the episode further contributed to the myth of the poisoner king.

To this day, “mad honey” poisonings continue in the Black Sea region, emphasizing the impact that environmental health can have not just on military operations but on all facets of human life.
